# Asymptotic behaviour of Stokes flow in a thin domain with a moving rough boundary

**DOI:** 10.1098/rspa.2013.0735

**Published:** 2014-07-08

**Authors:** J. Fabricius, Y. O. Koroleva, A. Tsandzana, P. Wall

**Affiliations:** 1 Department of Engineering Sciences and Mathematics, Luleå University of Technology, Luleå 971 87, Sweden; 2 Faculty of Mechanics and Mathematics, Department of Differential Equations, Moscow Lomonosov State University, Moscow 119991, Russia; 3 Faculty of Science, Department of Computer Science and Mathematics, Eduardo Mondlane University, Maputo, Mozambique

**Keywords:** lubrication theory, Reynolds equation, surface roughness, homogenization theory

## Abstract

We consider a problem that models fluid flow in a thin domain bounded by two surfaces. One of the surfaces is rough and moving, whereas the other is flat and stationary. The problem involves two small parameters *ϵ* and *μ* that describe film thickness and roughness wavelength, respectively. Depending on the ratio λ=*ϵ*/*μ*, three different flow regimes are obtained in the limit as both of them tend to zero. Time-dependent equations of Reynolds type are obtained in all three cases (Stokes roughness, Reynolds roughness and high-frequency roughness regime). The derivations of the limiting equations are based on formal expansions in the parameters *ϵ* and *μ*.

## 1. Introduction

The fundamental problem in lubrication theory is to describe fluid flow in a gap between two adjacent surfaces which are in relative motion. In the incompressible case, the main unknown is the pressure of the fluid. Having resolved the pressure it is possible to compute other fundamental quantities such as the velocity field and the forces on the bounding surfaces. To increase the hydrodynamic performance in various lubricated machine elements, for example journal bearings and thrust bearings, it is important to understand the influence of surface roughness. In this connection, one encounters various approaches, commonly based on the equation proposed by Osborne Reynolds in 1886 [1]. Although a number of averaging methods considering surfaces roughness have been proposed over the last 40 years (e.g. [2–4]), homogenization has prevailed as the proper way to average [5,6]. Homogenization is a rigorous mathematical theory that takes into account information about local effects on the microscopic level [7].

This study is concerned with the asymptotic behaviour of Stokes flow in a narrow gap described by two small parameters *ϵ* and *μ*. The parameter *ϵ* is related to the distance between the surface, whereas *μ* is the wavelength of the periodic roughness. In many problems involving two small parameters, the way in which the parameters tend to zero is primordial and the limiting equations may be different whether *ϵ* tends to zero faster, slower or at the same rate as *μ*. Using formal asymptotic expansions in the evolution Stokes equations, we show that three different asymptotic solutions, i.e. three different flow regimes, exist in the limit as *ϵ*>0 and *μ*>0 tend to zero depending on whether the limiting ratio

$$\lambda =\lim_{\left(\epsilon ,\mu )\to (0,0\right)}\frac{\epsilon }{\mu }$$

equals zero, a positive number or ∞. In all three flow regimes, the limiting pressure is governed by a two-dimensional equation of Reynolds type whose coefficients take into account the fine microstructure of the surface, i.e. a homogenized equation. The situation can be summarized as follows:

*Stokes roughness regime.* The case when 0<λ<∞. One finds that the coefficients of the homogenized equation are obtained by solving three-dimensional so-called cell problems which depend on the parameter λ.
*Reynolds roughness regime.* The case when λ=0. The cell problems are two-dimensional and the proposed averaged equation appears in, for example, [3,8,9]. The same limiting equations are obtained if one lets λ→0 in the Stokes roughness.
*High-frequency roughness regime.* The case when λ=∞. We obtain a limiting equation of very easy and cheap treatment. The same limiting equations are obtained if one lets λ→∞ in the Stokes roughness.

This work is closely related to the studies by Bayada & Chambat [4,10] and Benhaboucha *et al.* [11], who considered the stationary case, i.e. only the flat surface is moving. The main novelty is the treatment of the unstationary case (the rough surface is moving) as well as the way that *ϵ* and *μ* tend to zero. The paper is organized as follows: §2 is devoted to the formulation of the problem and basic notations. Section 3 contains a summary of the main results of this work. In §4, we define the formal asymptotic expansions, the corresponding change of variables, domains and differential operators for the problem. Section 5 is concerned with the Stokes roughness, with constant ratio *ϵ*/*μ*=λ. This is the case analysed in [4,10]. Section 6 is devoted to the case *ϵ*=*μ*^2^, which corresponds to Reynolds roughness. We apply the asymptotic expansion method in one parameter and derive the homogenized Reynolds equations. The last section deals with the case *μ*=*ϵ*^2^, which belongs to the high-frequency roughness regime. We obtain the classical Reynolds equations with truncated film thickness. We note that neither *ϵ*=*μ*^2^ nor *μ*=*ϵ*^2^ is covered in [4,10], whereas [11] only covers *ϵ*=*μ*^2^. Evidently, as mentioned in [4,10], identical equations are obtained if one lets λ→0 and λ→∞ in the Stokes roughness regime. However, from a mathematical point of view, there is no apparent reason why taking limits in such different ways would yield the same result.

For clarity, the main results are presented as ‘theorems’ and their derivations as ‘proofs’, although the method of formal expansion is not rigorous by mathematical standards. Choosing this style, we hope to make the paper accessible to a wider audience. We stress, however, that all calculations (including limit processes) can be made rigorous.

## 2. Problem formulation and basic notations

This study is concerned with thin film hydrodynamic lubrication of rough surfaces. For simplicity, we suppose that one of the surfaces is rough and moves with velocity *v*=(*v*_1_,*v*_2_,0) and that the other is flat and stationary. As the rough surface is moving, the film thickness varies in both space and time, thus rendering the problem unstationary. A point in space ($R^{3}$) is denoted as *x*=(*x*_1_,*x*_2_,*x*_3_), and *t* is a time variable that belongs to the interval [0,*T*]. The problem considered is the evolution Stokes system

2.1 $$\frac{\partial u}{\partial t}-\nu \Delta u+\nabla p=0$$

and

2.2 div u=0,

where *ν* (viscosity) is a constant, and *u*=(*u*_1_,*u*_2_,*u*_3_) (velocity field) and *p* (pressure) are unknown.

We shall write

2.3 $$x^{\prime }=(x_{1},x_{2}),\;z=\frac{x_{3}}{\epsilon },\;y^{\prime }=\frac{x^{\prime }}{\mu }\;\text{and}\;\tau =\frac{t}{\mu },$$

where *ϵ* and *μ* are two small parameters. The basic idea of the homogenization method is to treat *x*′,*y*′,*t* and *τ* as independent variables. Equations (2.1) and (2.2) are assumed to hold in a moving space domain *Ω*_*ϵμ*_(*t*), defined by

$$\Omega_{\epsilon \mu }(t)=\left\{x=(x_{1},x_{2},x_{3})\in R^{3},\;x^{\prime }=(x_{1},x_{2})\in \omega ,\;0<x_{3}<\epsilon H\left(x^{\prime },\frac{x^{\prime }}{\mu },t,\frac{t}{\mu }\right)\right\},$$

where *ω* is an open connected set in $R^{2}$ with smooth boundary, outward unit normally denoted by $\hat{n}$ and the function *H*(*x*′,*y*′,*t*,*τ*) describes the geometry of the upper surface. *H* is assumed to be *Y* -periodic in *y*′, *Y* =[0,1]×[0,1] being the cell of periodicity and T-periodic in *τ*. More precisely,

$$H(x^{\prime },y^{\prime },t,\tau )=h_{0}(x^{\prime }-tv)+h_{\mathrm{per}}(y^{\prime }-\tau v),$$

where *h*_0_ describes the global film thickness, whereas the *Y* -periodic function *h*_per_ represents the roughness. Thus, *ϵ* is related to the film thickness, whereas *μ* is the wavelength of the roughness. Moreover, we define the ‘minimum film thickness’

$$H^{*}(x^{\prime },t)=h_{0}(x^{\prime }-tv)+\min_{y^{\prime }\in Y}h_{\mathrm{per}}(y^{\prime }).$$

The boundaries of *Ω*_*ϵμ*_(*t*) are

$$\begin{array}{rl} \Sigma_{\epsilon \mu }^{+}(t) & =\left\{x\in R^{3}:x^{\prime }=(x_{1},x_{2})\in \omega ,\;x_{3}=\epsilon H\left(x^{\prime },\frac{x^{\prime }}{\mu },t,\frac{t}{\mu }\right)\right\},\\ \Sigma_{\epsilon \mu }^{-}(t) & =\{x\in R^{3}:x^{\prime }=(x_{1},x_{2})\in \omega ,\;x_{3}=0\}\\ \mathrm{and}\;\Sigma_{\epsilon \mu }^{w}(t) & =\left\{x\in R^{3}:x^{\prime }=(x_{1},x_{2})\in \partial \omega ,\;0\leq x_{3}\leq \epsilon H\left(x^{\prime },\frac{x^{\prime }}{\mu },t,\frac{t}{\mu }\right)\right\}. \end{array}$$

We assume the following no-slip boundary conditions:

$$\begin{array}{rl} u & =\left(v_{1},v_{2},\epsilon \left(\frac{\partial H}{\partial t}+v\cdot \nabla H\right)\right)\;\text{on}\;\Sigma_{\epsilon \mu }^{+}(t),\\ u & =0\;\;\;\text{on}\;\Sigma_{\epsilon \mu }^{-}(t)\\ \mathrm{and}\;\;u & =\left(\begin{array}{c} g_{1}\left(x_{1},x_{2},\frac{x_{3}}{\epsilon }\right)\\ g_{2}\left(x_{1},x_{2},\frac{x_{3}}{\epsilon }\right)\\ \epsilon g_{3}\left(x_{1},x_{2},\frac{x_{3}}{\epsilon }\right) \end{array}\right)\;\text{on}\;\Sigma_{\epsilon \mu }^{w}(t), \end{array}$$

where *g*=(*g*_1_,*g*_2_,*g*_3_) is some given function and the initial condition

$$u=\left(\begin{array}{c} U_{1}^{0}\left(x_{1},x_{2},\frac{x_{3}}{\epsilon }\right)\\ U_{2}^{0}\left(x_{1},x_{2},\frac{x_{3}}{\epsilon }\right)\\ \epsilon U_{3}^{0}\left(x_{1},x_{2},\frac{x_{3}}{\epsilon }\right) \end{array}\right)\;\text{on }\Omega_{\epsilon \mu }(0)\times \{0\},$$

where ∇*H*=(∂*H*/∂*x*_1_,∂*H*/∂*x*_2_,0).

For convenience, we use the notation

$${\bar{f}}^{y^{\prime }}=\int_{Y}f\;dy^{\prime },\;{\bar{f}}^{z}=\int_{0}^{H}f\;dz\;\text{and}\;{\bar{f}}^{z*}=\int_{0}^{H^{*}}f\;dz$$

for integrals of a function *f*. Moreover, we denote by *e*_1_,*e*_2_,*e*_3_ the standard basis vectors in $R^{3}.$

Finally, to ensure the existence of *u*, we must require some compatibility between the boundary conditions and *H*. To this end, it is assumed that *g* is a *C*^1^ vector field defined on $R^{3}$ such that div*g*=0, *g*(*x*_1_,*x*_2_,0)=(0,0,0),

$$g_{1}(x_{1},x_{2},H)=v_{1},\;g_{2}(x_{1},x_{2},H)=v_{2}\;\mathrm{and}\;g_{3}(x_{1},x_{2},H)=\frac{\partial H}{\partial t}+v\cdot \nabla H$$

and

2.4 $$\int_{\omega }\frac{\partial H}{\partial t}\;dx^{\prime }+\int_{\partial \omega }\left(\int_{0}^{H}g\;dz\right)\cdot \hat{n}\;dS=0$$

for all (*y*′,*t*,*τ*).

## 3. Formal asymptotic expansion in *ϵ* and *μ*

We analyse the asymptotic behaviour of the equations of motion (2.1) and (2.2). We define the following expansions for *u* and *p*:

$$u(x,t)=\sum_{n=0}^{\infty }\sum_{m=0}^{\infty }\varepsilon^{n}\mu^{m}u^{n,m}(x^{\prime },z,y^{\prime },t,\tau )$$

and

$$p(x,t)=\sum_{n=-2}^{\infty }\sum_{m=0}^{\infty }\varepsilon^{n}\mu^{m}p^{n,m}(x^{\prime },z,y^{\prime },t,\tau ),$$

where *x*′,*z*,*y*′ and *τ* are defined by (2.3) though subsequently treated as independent variables. As the roughness is periodic, it is assumed that *u*^*n*,*m*^(*x*′,*z*,*y*′,*t*,*τ*) and *p*^*n*,*m*^(*x*′,*z*,*y*′,*t*,*τ*) are *Y* -periodic in *y*′ and T-periodic in *τ*. It is convenient to define also the following domains:

$$\begin{array}{lll} \Omega (y',t,\tau ) & = & \left\{(x',z)\in \omega \times \mathbb{R}:0<z<H(x',y',t,\tau )\right\}\\ \text{B}(x',t,\tau ) & = & \left\{(y',z)\in Y\times \mathbb{R}:0<z<H(x',y',t,\tau )\right\}\\ {\text{B}}_{H*}^{+}(x',t,\tau ) & = & \left\{(y',z)\in Y\times \mathbb{R}:H*(x',t)<z<H(x',y',t,\tau )\right\}\\ \Omega *(t) & = & \left\{(x',z)\in \omega \times \mathbb{R}:0<z<H*(x',t)\right\}\\ \text{B*}(x',t) & = & \left\{(y',z)\in Y\times \mathbb{R}:0<z<H*(x',t)\right\}\\ Y_{z}(x',t,\tau ) & = & \{y'\in Y:(y',z)\in \text{B}(x',t,\tau )\}. \end{array}$$

The boundaries of *Ω*(*y*′,*t*,*τ*) and Ω*(*t*) are

$$\begin{array}{rl} \Sigma^{+}(y^{\prime },t,\tau ) & =\{(x^{\prime },z)\in \omega \times R:z=H(x^{\prime },y^{\prime },t,\tau )\},\\ \Sigma^{-}(y^{\prime },t,\tau ) & =\{(x^{\prime },z)\in \omega \times R:z=0\},\\ \Sigma^{w}(y^{\prime },t,\tau ) & =\{(x^{\prime },z)\in \partial \omega \times R:0\leq z\leq H(x^{\prime },y^{\prime },t,\tau )\},\\ \Sigma^{*+}(t) & =\{(x^{\prime },z)\in \omega \times R:z=H^{*}(x^{\prime },t)\},\\ \Sigma^{*-}(t) & =\{(x^{\prime },z)\in \omega \times R:z=0\}\\ \mathrm{and}\;\;\Sigma^{*w}(t) & =\{(x^{\prime },z)\in \partial \omega \times R:0\leq z\leq H^{*}(x^{\prime },t)\}. \end{array}$$

Note that B(*x*′,*t*,*τ*) and B*(*x*′,*t*) do not have lateral boundaries because of the periodicity of *Y* . The boundary of *Y*_*z*_ is denoted by

$$\partial Y_{z}(x^{\prime },t,\tau )=\{y^{\prime }\in Y:z=H(x^{\prime },y^{\prime },t,\tau )\}.$$

### (a) Differential operators

Assume *u*(*x*,*t*)=*v*(*x*′,*z*,*y*′,*t*,*τ*). Then,

$$\begin{array}{rl} \frac{\partial u}{\partial t} & =\frac{\partial v}{\partial t}+\frac{1}{\mu }\frac{\partial v}{\partial \tau },\\ \nabla u & =\left(\frac{\partial v}{\partial x_{1}},\frac{\partial v}{\partial x_{2}},0\right)+\frac{1}{\mu }\left(\frac{\partial v}{\partial y_{1}},\frac{\partial v}{\partial y_{2}},0\right)+\frac{1}{\epsilon }\left(0,0,\frac{\partial v}{\partial z}\right)\\ \mathrm{and}\;\;\Delta u & =\left(\frac{\partial^{2}}{\partial x_{1}^{2}}+\frac{\partial^{2}}{\partial x_{2}^{2}}\right)v+\frac{2}{\mu }\left(\frac{\partial^{2}}{\partial x_{1}\partial y_{1}}+\frac{\partial^{2}}{\partial x_{2}\partial y_{2}}\right)v+\frac{1}{\mu^{2}}\left(\frac{\partial^{2}}{\partial y_{1}^{2}}+\frac{\partial^{2}}{\partial y_{2}^{2}}\right)v+\frac{1}{\epsilon^{2}}\left(\frac{\partial^{2}}{\partial z^{2}}\right)v. \end{array}$$

Define

$$\nabla_{x^{\prime }}=\left(\frac{\partial }{\partial x_{1}},\frac{\partial }{\partial x_{2}},0\right),\;\nabla_{y^{\prime }}=\left(\frac{\partial }{\partial y_{1}},\frac{\partial }{\partial y_{2}},0\right)\;\mathrm{and}\;\nabla_{z}=\left(0,0,\frac{\partial }{\partial z}\right)$$

and

$$\Delta_{x^{\prime }}=\frac{\partial^{2}}{\partial x_{1}^{2}}+\frac{\partial^{2}}{\partial x_{2}^{2}},\;\Delta_{y^{\prime }}=\frac{\partial^{2}}{\partial y_{1}^{2}}+\frac{\partial^{2}}{\partial y_{2}^{2}}\;\mathrm{and}\;\Delta_{z}=\frac{\partial^{2}}{\partial z^{2}}.$$

Moreover,

$${\mathrm{div}}_{x^{\prime }}v=\nabla_{x^{\prime }}\cdot v\;\text{and}\;{\mathrm{div}}_{y^{\prime }}v=\nabla_{y^{\prime }}\cdot v.$$

## 4. Stokes roughness

**Figure 1.**
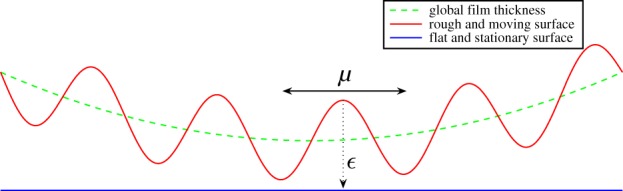
Stokes roughness (*μ*=*ϵ*/λ). (Online version in colour.)

 Figure 1 describes the case when *ϵ* and *μ* tend to zero with constant ratio 0<λ<∞. That is, we assume that *μ*=*ϵ*/λ, where λ is a positive constant. We define the following asymptotic expansions:

4.1 $$u(x,t)=\sum_{n=0}^{\infty }\epsilon^{n}u^{n}(x^{\prime },z,y^{\prime },t,\tau )$$

and

4.2 $$p(x,t)=\sum_{n=-2}^{\infty }\epsilon^{n}p^{n}(x^{\prime },z,y^{\prime },t,\tau ).$$

Inserting (4.1) and (4.2) into (2.1) and equating terms of the same order using

$$\begin{array}{rl} \frac{\partial }{\partial t} & \to \frac{\partial }{\partial t}+\frac{\lambda }{\epsilon }\frac{\partial }{\partial \tau },\\ \nabla & \to \nabla_{x^{\prime }}+\frac{\lambda }{\epsilon }\nabla_{y^{\prime }}+\frac{1}{\epsilon }\nabla_{z}\\ \mathrm{and}\;\;\Delta & \to \Delta_{x^{\prime }}+\frac{2\lambda }{\epsilon }\left(\frac{\partial^{2}}{\partial x_{1}\partial y_{1}}+\frac{\partial^{2}}{\partial x_{2}\partial y_{2}}\right)+\frac{\lambda^{2}}{\epsilon^{2}}\Delta_{y^{\prime }}+\frac{1}{\epsilon^{2}}\Delta_{z} \end{array}$$

gives

4.3 $$\begin{array}{r} \frac{1}{\epsilon^{3}}:\lambda \nabla_{y^{\prime }}p^{-2}+\nabla_{z}p^{-2}=0, \end{array}$$

4.4 $$\begin{array}{r} \frac{1}{\epsilon^{2}}:-\nu \lambda^{2}\Delta_{y^{\prime }}u^{0}-\nu \Delta_{z}u^{0}+\nabla_{x^{\prime }}p^{-2}+\lambda \nabla_{y^{\prime }}p^{-1}+\nabla_{z}p^{-1}=0 \end{array}$$

4.5 $$\begin{array}{c} \mathrm{and}\;\frac{1}{\epsilon }:\lambda \frac{\partial u^{0}}{\partial \tau }-\nu \left(2\lambda \frac{\partial^{2}u^{0}}{\partial x_{1}\partial y_{1}}+2\lambda \frac{\partial^{2}u^{0}}{\partial x_{2}\partial y_{2}}+\Delta_{z}u^{1}+\lambda^{2}\Delta_{y^{\prime }}u^{1}\right)\\ +\nabla_{x^{\prime }}p^{-1}+\lambda \nabla_{y^{\prime }}p^{0}+\nabla_{z}p^{0}=0. \end{array}$$

Similarly for (2.2), we have

4.6 $$\frac{1}{\epsilon }:\lambda \;{\mathrm{div}}_{y^{\prime }}u^{0}+\frac{\partial u_{3}^{0}}{\partial z}=0$$

and

4.7 $$\epsilon^{0}:{\mathrm{div}}_{x^{\prime }}u^{0}+\lambda \;{\mathrm{div}}_{y^{\prime }}u^{1}+\frac{\partial u_{3}^{1}}{\partial z}=0.$$

The boundary conditions are

$$\begin{array}{rl} \epsilon^{0}:u^{0} & =\left\{ \begin{array}{ll} \left(v_{1},v_{2},\lambda \left(\frac{\partial H}{\partial \tau }+v\cdot \nabla_{y^{\prime }}H\right)\right) & \text{on }\Sigma^{+}\\ 0 & \text{on }\Sigma^{-}\\ \left(g_{1},g_{2},0\right) & \text{on }\Sigma^{w} \end{array}\right.\\ \mathrm{and}\;\;\epsilon^{1}:u^{1} & =\left\{ \begin{array}{ll} \left(0,0,\frac{\partial H}{\partial t}+v\cdot \nabla_{x^{\prime }}H\right) & \text{on }\Sigma^{+}\\ 0 & \text{on }\Sigma^{-}\\ \left(0,0,g_{3}\right) & \text{on }\Sigma^{w} \end{array}\right. \end{array}$$

and initial conditions are

$$\begin{array}{rl} \epsilon^{0}:u^{0} & =(U_{1}^{0},U_{2}^{0},0)\;\text{on }\Omega \times \{t=0\}\\ \mathrm{and}\;\;\epsilon^{1}:u^{1} & =(0,0,U_{3}^{0})\;\text{on }\Omega \times \{t=0\}. \end{array}$$

### (a) Analysis of equations

The main result pertaining to the Stokes roughness is as follows.

**Theorem 4.1**

*The leading term u*^0^ *in expansion* (4.1) *for u is given by*

4.8 $$u^{0}=\sum_{i=1}^{2}\frac{\partial p^{-2}}{\partial x_{i}}\alpha^{i}+\alpha^{0},$$

*where α*^*i*^*(i=0,1,2) is a solution of the periodic cell problems* (4.12) *and* (4.13) *and the leading term p*^−2^ *in expansion* (4.2) *for ϵ*^2^*p is a solution of the boundary value problem*

4.9 $${\mathrm{div}}_{x^{\prime }}(A^{\lambda }\nabla_{x^{\prime }}p^{-2}+b^{\lambda })+\frac{\partial {\bar{H}}^{y^{\prime }}}{\partial t}=0\;\text{in }\omega \times (0,T]$$

*and*

4.10 $$(A^{\lambda }\nabla_{x^{\prime }}p^{-2}+b^{\lambda }-{\bar{{\bar{g}}^{z}}}^{y^{\prime }})\cdot \hat{n}=0\;\text{on }\partial \omega \times (0,T],$$

*where A*^λ^ *and b*^λ^ *are calculated by* (4.17) *and* (4.18), *respectively.*

**Proof.**

If we write (4.3) in component form, we obtain

$$\frac{\partial p^{-2}}{\partial y_{1}}=0,\;\frac{\partial p^{-2}}{\partial y_{2}}=0\;\text{and}\;\frac{\partial p^{-2}}{\partial z}=0,$$

hence *p*^−2^(*x*′,*y*′,*z*,*t*,*τ*)=*p*^−2^(*x*′,*t*,*τ*). We are looking for solutions *u*^0^ of the form (4.8) and *p*^−1^ of the form

4.11 $$p^{-1}=\sum_{i=1}^{2}\frac{\partial p^{-2}}{\partial x_{i}}q^{i}+q^{0},$$

where *α*^*i*^=*α*^*i*^(*x*′,*z*,*y*′,*t*,*τ*) and *q*^*i*^=*q*^*i*^(*x*′,*z*,*y*′,*t*,*τ*) are to be determined. Clearly, (4.8) and (4.11) satisfy (4.4) and (4.6) if

4.12 $$\nu \lambda^{2}\Delta_{y^{\prime }}\alpha^{i}+\nu \Delta_{z}\alpha^{i}=\lambda \nabla_{y^{\prime }}q^{i}+\nabla_{z}q^{i}+e_{i}\;\text{in }B\;(i=0,1,2)$$

and

4.13 $$\lambda \;{\mathrm{div}}_{y^{\prime }}\alpha^{i}+\frac{\partial \alpha_{3}^{i}}{\partial z}=0\;\text{in }B\;(i=0,1,2).$$

The above systems of equations are called cell problems, whose solutions *α*^*i*^ and *q*^*i*^ are *Y* -periodic, and the boundary conditions are

$$\begin{array}{rl} \alpha^{i} & =\left\{ \begin{array}{ll} \left(v_{1},v_{2},\lambda \left(\frac{\partial H}{\partial \tau }+v\cdot \nabla_{y^{\prime }}H\right)\right), & \left(i=0\right)\\ 0, & \left(i=1,2\right) \end{array}\right.\;\text{on }S^{+}\\ \mathrm{and}\;\;\alpha^{i} & =0,\;(i=0,1,2)\;\text{on }S^{-}. \end{array}$$

It can be verified that each *α*^*i*^ is uniquely determined by (4.12) and (4.13).

Multiplying (4.7) by $\phi (x^{\prime })\in C^{1}(\bar{\omega })$ and integrating by parts using the Gauss–Green theorem, we obtain

$$\begin{array}{rl} 0 & =\int_{\omega }\int_{Y}\int_{0}^{H}\left({\mathrm{div}}_{x^{\prime }}u^{0}+\lambda \;{\mathrm{div}}_{y^{\prime }}u^{1}+\frac{\partial u_{3}^{1}}{\partial z}\right)\phi (x^{\prime })\;dz\;dy^{\prime }\;dx^{\prime }\\ & =\int_{Y}\int_{\Omega }{\mathrm{div}}_{x^{\prime }}u^{0}\phi \;dx^{\prime }\;dz\;dy^{\prime }+\int_{\omega }\int_{B}\left(\lambda \;{\mathrm{div}}_{y^{\prime }}u^{1}+\frac{\partial u_{3}^{1}}{\partial z}\right)\phi \;dy^{\prime }\;dz\;dx^{\prime }\\ & =\int_{Y}\int_{\Omega }-u^{0}\cdot \nabla_{x^{\prime }}\phi \;dz\;dx^{\prime }\;dy^{\prime }+\int_{Y}\int_{\Sigma^{+}}\phi (u_{1}^{0},u_{2}^{0},u_{3}^{1})\cdot \hat{n}\;dS(x^{\prime },z)\;dy^{\prime }\\ & \;+\int_{Y}\int_{\Sigma^{w}}\phi g\cdot \hat{n}\;dS(x^{\prime },z)\;dy+\int_{\omega }\int_{\partial B}\phi \lambda (u_{1}^{1},u_{2}^{1},0)\cdot \hat{n}\;dS(y^{\prime },z)\;dx^{\prime }\\ & =\int_{Y}\int_{\omega }-{\bar{u^{0}}}^{z}\cdot \nabla_{x^{\prime }}\phi dy^{\prime }\;dx^{\prime }+\int_{Y}\int_{\partial \omega }\int_{0}^{H}\phi g\cdot ({\hat{n}}_{1},{\hat{n}}_{2},0)\;dS(x^{\prime },z)\;dy\\ & \;+\int_{Y}\int_{\omega }-v\cdot \nabla_{x^{\prime }}H\phi +\left(\frac{\partial H}{\partial t}+v\cdot \nabla_{x^{\prime }}H\right)\phi \;dx^{\prime }\;dy\\ & =\int_{\omega }-{\bar{{\bar{u^{0}}}^{z}}}^{y^{\prime }}\cdot \nabla_{x^{\prime }}\phi +\frac{\partial {\bar{H}}^{y^{\prime }}}{\partial t}\phi \;dx^{\prime }+\int_{\partial \omega }\phi {\bar{{\bar{g}}^{z}}}^{y^{\prime }}\cdot \hat{n}\;dS(x^{\prime }). \end{array}$$

As *ϕ* is arbitrary, it holds that

4.14 $${\mathrm{div}}_{x^{\prime }}{\bar{{\bar{u^{0}}}^{z}}}^{y^{\prime }}+\frac{\partial {\bar{H}}^{y^{\prime }}}{\partial t}=0\;\text{in }\omega \times (0,T]$$

and

4.15 $$({\bar{{\bar{u^{0}}}^{z}}}^{y^{\prime }}-{\bar{{\bar{g}}^{z}}}^{y^{\prime }})\cdot \hat{n}=0\;\text{on }\partial \omega \times (0,T].$$

By integrating (4.8), we obtain

4.16 $${\bar{{\bar{u^{0}}}^{z}}}^{y^{\prime }}=\sum_{i=1}^{2}\frac{\partial p^{-2}}{\partial x_{i}}{\bar{{\bar{\alpha^{i}}}^{z}}}^{y^{\prime }}+{\bar{{\bar{\alpha^{0}}}^{z}}}^{y^{\prime }}=A^{\lambda }\nabla_{x^{\prime }}p^{-2}+b^{\lambda },$$

where

4.17 $$A^{\lambda }={\bar{{\bar{\left(\begin{array}{ccc} \alpha_{1}^{1} & \alpha_{1}^{2} & 0\\ \alpha_{2}^{1} & \alpha_{2}^{2} & 0\\ \alpha_{3}^{1} & \alpha_{3}^{2} & 0 \end{array}\right)}}^{z}}}^{y^{\prime }}$$

and

4.18 $$b^{\lambda }={\bar{{\bar{\left(\begin{array}{c} \alpha_{1}^{0}\\ \alpha_{2}^{0}\\ \alpha_{3}^{0} \end{array}\right)}}^{z}}}^{y^{\prime }}.$$

Inserting (4.16) into (4.14) and (4.15), we obtain the homogenized Reynolds equation (4.9) with the boundary (4.10). □

## 5. Reynolds roughness

**Figure 2.**
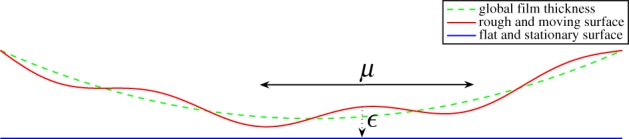
Reynolds roughness (*μ*≫*ϵ*). (Online version in colour.)

 Figure 2 describes the case when the wavelength of the roughness is much greater than the film thickness, i.e. *μ*≫*ϵ*. This case can be studied by assuming that *ϵ* is a function of *μ* such that

$$\lim_{\mu \to 0}\frac{\varepsilon (\mu )}{\mu }=0.$$

For simplicity, we shall assume that *ϵ*=*μ*^2^. We postulate the following expansions for *u* and *p*:

5.1 $$u(x,t)=\sum_{n=0}^{\infty }\mu^{n}u^{n}(x^{\prime },z,y^{\prime },t,\tau )$$

and

5.2 $$p(x,t)=\sum_{n=-4}^{\infty }\mu^{n}p^{n}(x^{\prime },z,y^{\prime },t,\tau ).$$

Plugging (5.1) and (5.2) into (2.1) and equating terms of the same order using

$$\begin{array}{rl} \frac{\partial }{\partial t} & \to \frac{\partial }{\partial t}+\frac{1}{\mu }\frac{\partial }{\partial \tau },\\ \nabla & \to \nabla_{x^{\prime }}+\frac{1}{\mu }\nabla_{y^{\prime }}+\frac{1}{\mu^{2}}\nabla_{z}\\ \mathrm{and}\;\;\Delta & \to \Delta_{x^{\prime }}+\frac{2}{\mu }\left(\frac{\partial^{2}}{\partial x_{1}\partial y_{1}}+\frac{\partial^{2}}{\partial x_{2}\partial y_{2}}\right)+\frac{1}{\mu^{2}}\Delta_{y^{\prime }}+\frac{1}{\mu^{4}}\Delta_{z} \end{array}$$

gives

5.3 $$\begin{array}{rl} & \frac{1}{\mu^{6}}:\nabla_{z}p^{-4}=0, \end{array}$$

5.4 $$\begin{array}{r} \frac{1}{\mu^{5}}:\nabla_{y^{\prime }}p^{-4}+\nabla_{z}p^{-3}=0 \end{array}$$

5.5 $$\begin{array}{r} \mathrm{and}\;\;\frac{1}{\mu^{4}}:-\nu \frac{\partial^{2}u^{0}}{\partial z^{2}}+\nabla_{x^{\prime }}p^{-4}+\nabla_{y^{\prime }}p^{-3}+\nabla_{z}p^{-2}=0. \end{array}$$

Similarly for (2.2), we have

5.6 $$\begin{array}{r} \frac{1}{\mu^{2}}:\frac{\partial u_{3}^{0}}{\partial z}=0, \end{array}$$

5.7 $$\begin{array}{r} \frac{1}{\mu }:{\mathrm{div}}_{y^{\prime }}u^{0}+\frac{\partial u_{3}^{1}}{\partial z}=0 \end{array}$$

5.8 $$\begin{array}{r} \mathrm{and}\;\;\mu^{0}:{\mathrm{div}}_{x^{\prime }}u^{0}+{\mathrm{div}}_{y^{\prime }}u^{1}+\frac{\partial u_{3}^{2}}{\partial z}=0. \end{array}$$

The boundaries conditions are

$$\begin{array}{rl} \mu^{0}:u^{0} & =\left\{ \begin{array}{ll} \left(v_{1},v_{2},0\right) & \text{on }\Sigma^{+}\\ 0 & \text{on }\Sigma^{-}\\ \left(g_{1},g_{2},0\right) & \text{on }\Sigma^{w}, \end{array}\right.\\ \mu^{1}:u^{1} & =\left\{ \begin{array}{ll} \left(0,0,\frac{\partial H}{\partial \tau }+v\cdot \nabla_{y^{\prime }}H\right) & \text{on }\Sigma^{+}\\ 0 & \text{on }\Sigma^{-}\\ 0 & \text{on }\Sigma^{w} \end{array}\right.\\ \mathrm{and}\;\;\;\mu^{2}:u^{2} & =\left\{ \begin{array}{ll} \left(0,0,\frac{\partial H}{\partial t}+v\cdot \nabla_{x^{\prime }}H\right) & \text{on }\Sigma^{+}\\ 0 & \text{on }\Sigma^{-}\\ \left(0,0,g_{3}\right) & \text{on }\Sigma^{w} \end{array}\right. \end{array}$$

and initial conditions are

$$\begin{array}{rl} \mu^{0}:u^{0} & =(u_{1}^{0},u_{2}^{0},0)\;\text{on }\underset{y^{\prime }\in Y}{⋃}\Omega_{y^{\prime },0,\mu }\times \{y^{\prime }\}\times \{0\},\\ \mu^{1}:u^{1} & =0\\ \mathrm{and}\;\mu^{2}:u^{2} & =(0,0,u_{3}^{0}). \end{array}$$

### (a) Analysis of equations

The main result is as follows.

**Theorem 5.1**

*The leading term u*^0^ *in expansion* (5.1) *for u is given by*

5.9 $$u^{0}=\frac{z(z-H)}{2\nu }(\nabla_{x^{\prime }}p^{-4}+\nabla_{y^{\prime }}p^{-3})+\frac{z}{H}v,$$

*where p*^−4^*, the leading term in expansion* (5.2) *for μ*^4^*p, is a solution of the boundary value problem*

5.10 $${\mathrm{div}}_{x^{\prime }}(-A\nabla_{x^{\prime }}p^{-4}+b)+\frac{\partial {\bar{H}}^{y^{\prime }}}{\partial t}=0\;\text{in }\omega$$

*and*

5.11 $$(-A\nabla_{x^{\prime }}p^{-4}+b-{\bar{{\bar{g}}^{z}}}^{y^{\prime }})\cdot \hat{n}=0\;\text{on }\partial \omega ,$$

*where A and b are given by* (5.22) *and* (5.23), *and*

5.12 $$p^{-3}=\sum_{i=1}^{2}\frac{\partial p^{-4}}{\partial x_{i}}q_{i}+q_{0},$$

*where q*_*i*_ *(i=0,1,2) is a periodic solution of the cell problems* (5.16) *and* (5.17).

**Proof.**

Equations (5.3) and (5.4) say that

$$\frac{\partial p^{-4}}{\partial z}=0,\;\frac{\partial p^{-4}}{\partial y_{1}}=0,\;\frac{\partial p^{-4}}{\partial y_{2}}=0\;\text{and }\;\frac{\partial p^{-3}}{\partial z}=0.$$

Therefore,

$$p^{-4}(x^{\prime },z,y^{\prime },t,\tau )=p^{-4}(x^{\prime },t,\tau )\;\text{and }\;p^{-3}(x^{\prime },z,y^{\prime },t,\tau )=p^{-3}(x^{\prime },y^{\prime },t,\tau ).$$

From (5.6), we deduce $u_{3}^{0}=0$ in *Ω* because of the boundary conditions for $u_{3}^{0}.$ Thus, (5.5) in component form becomes

$$\begin{array}{rl} -\nu \frac{\partial^{2}u_{1}^{0}}{\partial z^{2}}+\frac{\partial p^{-4}}{\partial x_{1}}+\frac{\partial p^{-3}}{\partial y_{1}} & =0,\\ -\nu \frac{\partial^{2}u_{2}^{0}}{\partial z^{2}}+\frac{\partial p^{-4}}{\partial x_{2}}+\frac{\partial p^{-3}}{\partial y_{2}} & =0\\ \mathrm{and}\;\;\frac{\partial p^{-2}}{\partial z} & =0. \end{array}$$

Hence, the first two equations may be written as

5.13 $$-\nu \frac{\partial^{2}u_{0}}{\partial z^{2}}+\nabla_{x^{\prime }}p^{-4}+\nabla_{y^{\prime }}p^{-3}=0.$$

Integrating (5.13) with respect to *z* and taking into account the boundary values of *u*^0^, we get (5.9). Integrating (5.9) once more, we obtain

5.14 $${\bar{u^{0}}}^{z}=-\frac{H^{3}}{12\nu }(\nabla_{x^{\prime }}p^{-4}+\nabla_{y^{\prime }}p^{-3})+\frac{H}{2}v.$$

Multiplying (5.7) with *ϕ*(*y*′) and integrating over B using the Gauss–Green theorem gives

$$\begin{array}{rl} 0 & =\int_{B}\left({\mathrm{div}}_{y^{\prime }}u^{0}+\frac{\partial u_{3}^{1}}{\partial z}\right)\phi (y^{\prime })\;dy^{\prime }\;dz\\ & =\int_{B}-u^{0}\cdot \nabla_{y^{\prime }}\phi \;dy^{\prime }\;dz+\int_{S^{+}}\phi (u_{1}^{0},u_{2}^{0},u_{3}^{1})\cdot \hat{n}\;dS(y^{\prime },z)\\ & =\int_{Y}\int_{0}^{H}-u^{0}\cdot \nabla_{y^{\prime }}\phi \;dz\;dy^{\prime }\\ & \;+\int_{Y}\phi \left(v_{1},v_{2},\frac{\partial H}{\partial \tau }+v\cdot \nabla_{y^{\prime }}H\right)\cdot \left(-\frac{\partial H}{\partial y_{1}},-\frac{\partial H}{\partial y_{2}},1\right)dy^{\prime }\;dz\\ & =\int_{Y}-{\bar{u^{0}}}^{z}\cdot \nabla_{y^{\prime }}\phi +\frac{\partial H}{\partial \tau }\phi \;dy^{\prime }\\ & =\int_{Y}\left({\mathrm{div}}_{y^{\prime }}{\bar{u^{0}}}^{z}+\frac{\partial H}{\partial \tau }\right)\phi \;dy^{\prime }, \end{array}$$

for all smooth and *Y* -periodic *ϕ*. Hence,

5.15 $${\mathrm{div}}_{y^{\prime }}{\bar{u^{0}}}^{z}+\frac{\partial H}{\partial \tau }=0,\;\text{in }Y.$$

Inserting (5.14) into (5.15), it is seen that *p*^−3^ can be written in the form (5.12), where *q*_*i*_ is periodic solutions of

5.16 $${\mathrm{div}}_{y^{\prime }}\left(-\frac{H^{3}}{12\nu }(\nabla_{y^{\prime }}q_{i}+e_{i})\right)=0,\;\text{in }Y\;(i=1,2)$$

and

5.17 $${\mathrm{div}}_{y^{\prime }}\left(-\frac{H^{3}}{12\nu }\nabla_{y^{\prime }}q_{0}+\frac{H}{2}v\right)+\frac{\partial H}{\partial \tau }=0,\;\text{in }Y.$$

This implies

5.18 $${\bar{u^{0}}}^{z}=-\frac{H^{3}}{12\nu }\left[\left(\begin{array}{cc} 1+\frac{\partial q_{1}}{\partial y_{1}} & \frac{\partial q_{2}}{\partial y_{1}}\\ \frac{\partial q_{1}}{\partial y_{2}} & 1+\frac{\partial q_{2}}{\partial y_{2}} \end{array}\right)\nabla_{x^{\prime }}p^{-4}+\nabla_{y^{\prime }}q_{0}\right]+\frac{H}{2}v.$$

Multiplying (5.8) with $\phi (x^{\prime })\in C^{1}(\bar{\omega })$ and integrating gives

$$\begin{array}{rl} 0 & =\int_{\omega }\int_{Y}\int_{0}^{H}\left({\mathrm{div}}_{x^{\prime }}u^{0}+{\mathrm{div}}_{y^{\prime }}u^{1}+\frac{\partial u_{3}^{2}}{\partial z}\right)\phi (x^{\prime })\;dz\;dy^{\prime }\;dx^{\prime }\\ & =\int_{Y}\int_{\Omega }{\mathrm{div}}_{x^{\prime }}u^{0}\phi \;dy^{\prime }\;dx^{\prime }+\int_{\omega }\int_{B}\left({\mathrm{div}}_{x^{\prime }}u^{1}+\frac{\partial u_{3}^{2}}{\partial z}\right)\phi \;dx^{\prime }\;dy^{\prime }\;dz\\ & =\int_{Y}\int_{\Omega }-u^{0}\cdot \nabla_{x^{\prime }}\phi \;dz\;dx^{\prime }\;dy^{\prime }+\int_{Y}\int_{\partial \Omega }\phi (u_{1}^{0},u_{2}^{0},u_{3}^{2})\cdot \hat{n}\;dS(x^{\prime },z)\;dy^{\prime }\\ & \;+\int_{\omega }\int_{\partial B}\phi (u_{1}^{1},u_{2}^{1},0)\cdot \hat{n}\;dS(y^{\prime },z)\\ & =\int_{Y}\int_{\Omega }\int_{0}^{H}-u^{0}\cdot \nabla_{x^{\prime }}\phi \;dz\;dx^{\prime }\;dy^{\prime }+\int_{Y}\int_{\Sigma^{w}}\phi g\cdot \hat{n}\;dS(x^{\prime },z)\;dy^{\prime }\\ & \;+\int_{Y}\int_{\Sigma^{+}}\phi \left(v_{1},v_{2},\frac{\partial H}{\partial t}+v\cdot \nabla_{x^{\prime }}H\right)\cdot \hat{n}\;dS(x^{\prime },z)\;dy^{\prime }\\ & =\int_{Y}\int_{\omega }-{\bar{u^{0}}}^{z}\cdot \nabla_{x^{\prime }}\phi \;dx^{\prime }\;dy^{\prime }+\int_{Y}\int_{\omega }\phi \left(-v\cdot \nabla_{x^{\prime }}+\frac{\partial H}{\partial t}+v\cdot \nabla_{x^{\prime }}H\right)dx^{\prime }\;dy^{\prime }\\ & \;+\int_{Y}\int_{\partial \omega }\int_{0}^{H}\phi g\cdot \hat{n}\;dS(x^{\prime },z)\;dy^{\prime }\\ & =\int_{\omega }-{\bar{{\bar{u^{0}}}^{z}}}^{y^{\prime }}\cdot \nabla_{x^{\prime }}\phi +\frac{\partial {\bar{H}}^{y^{\prime }}}{\partial t}\phi \;dx^{\prime }+\int_{\partial \omega }\phi {\bar{{\bar{g}}^{z}}}^{y^{\prime }}\cdot \hat{n}\;dS(x^{\prime },z). \end{array}$$

As *ϕ* is arbitrary, it holds that

5.19 $${\mathrm{div}}_{x^{\prime }}{\bar{{\bar{u^{0}}}^{z}}}^{y^{\prime }}+\frac{\partial {\bar{H}}^{y^{\prime }}}{\partial t}=0\;\text{in }\omega$$

and

5.20 $$({\bar{{\bar{u^{0}}}^{z}}}^{y^{\prime }}-{\bar{{\bar{g}}^{z}}}^{y^{\prime }})\cdot \hat{n}=0\;\text{on }\partial \omega ,$$

where

5.21 $${\bar{{\bar{u^{0}}}^{z}}}^{y^{\prime }}=A\nabla_{x^{\prime }}p^{-4}+b,$$

5.22 $$A=\int_{Y}\frac{H^{3}}{12\nu }\left(\begin{array}{ccc} 1+\frac{\partial q_{1}}{\partial y_{1}} & \frac{\partial q_{2}}{\partial y_{1}} & 0\\ \frac{\partial q_{1}}{\partial y_{2}} & 1+\frac{\partial q_{2}}{\partial y_{2}} & 0\\ 0 & 0 & 0 \end{array}\right)dy^{\prime }$$

and

5.23 $$b=\int_{Y}-\frac{H^{3}}{12\nu }\nabla_{y^{\prime }}q_{0}+\frac{H}{2}v\;dy^{\prime }.$$

Inserting (5.21) into (5.19) and (5.20), we obtain the homogenized Reynolds equation (5.10) with boundary condition (5.11). □

**Remark 5.2**

The equations of theorem 5.1 can also be obtained by letting λ→0 in the equations of theorem 4.1.

## 6. High-frequency roughness regime

**Figure 3.**
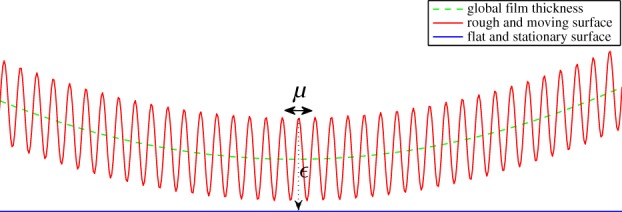
High-frequency roughness regime (*ϵ*≫*μ*). (Online version in colour.)

 Figure 3 illustrates the case when the roughness wavelength is small compared with the film thickness, i.e. *ϵ*≫*μ*. To this end, one assumes that *μ* is a function of *ϵ* such that

$$\lim_{\varepsilon \to 0}\frac{\epsilon }{\mu (\epsilon )}=\infty .$$

For simplicity, we shall assume *μ*=*ϵ*^2^. We postulate the following expansions:

6.1 $$u(x,t)=\sum_{n=0}^{\infty }\epsilon^{n}u^{n}(x^{\prime },z,y^{\prime },t,\tau )$$

and

6.2 $$p(x,t)=\sum_{n=-2}^{\infty }\epsilon^{n}p^{n}(x^{\prime },z,y^{\prime },t,\tau ).$$

Plug (6.1) and (6.2) into (2.1) and equating the terms of the same order using

$$\begin{array}{rl} \frac{\partial }{\partial t} & \to \frac{\partial }{\partial t}+\frac{1}{\epsilon^{2}}\frac{\partial }{\partial \tau },\\ \nabla & \to \nabla_{x^{\prime }}+\frac{1}{\epsilon^{2}}\nabla_{y^{\prime }}+\frac{1}{\epsilon }\nabla_{z}\\ \mathrm{and}\;\;\Delta & \to \Delta_{x^{\prime }}+\frac{2}{\epsilon^{2}}\left(\frac{\partial^{2}}{\partial x_{1}\partial y_{1}}+\frac{\partial^{2}}{\partial x_{2}\partial y_{2}}\right)+\frac{1}{\epsilon^{4}}\Delta_{y^{\prime }}+\frac{1}{\epsilon^{2}}\Delta_{z} \end{array}$$

that gives

6.3 $$\begin{array}{r} \frac{1}{\epsilon^{4}}:-\nu \Delta_{y^{\prime }}u^{0}+\nabla_{y^{\prime }}p^{-2}=0, \end{array}$$

6.4 $$\begin{array}{r} \frac{1}{\epsilon^{3}}:-\nu \Delta_{y^{\prime }}u^{1}+\nabla_{z}p^{-2}+\nabla_{y^{\prime }}p^{-1}=0 \end{array}$$

6.5 $$\begin{array}{rl} \mathrm{and}\;\; & \frac{1}{\epsilon^{2}}:\frac{\partial u^{0}}{\partial \tau }-\nu \left(2\frac{\partial^{2}u^{0}}{\partial x_{1}\partial y_{1}}+2\frac{\partial^{2}u^{0}}{\partial x_{2}\partial y_{2}}+\frac{\partial^{2}u^{0}}{\partial z^{2}}+\Delta_{y^{\prime }}u^{2}\right)\\ & \;+\nabla_{x^{\prime }}p^{-2}+\nabla_{z}p^{-1}+\nabla_{y^{\prime }}p^{0}=0. \end{array}$$

Similarly for (2.2), we have

6.6 $$\begin{array}{r} \frac{1}{\epsilon^{2}}:{\mathrm{div}}_{y^{\prime }}u^{0}=0, \end{array}$$

6.7 $$\begin{array}{r} \frac{1}{\epsilon }:{\mathrm{div}}_{y^{\prime }}u^{1}+\frac{\partial u_{3}^{0}}{\partial z}=0 \end{array}$$

6.8 $$\begin{array}{r} \mathrm{and}\;\;\epsilon^{0}:{\mathrm{div}}_{x^{\prime }}u^{0}+{\mathrm{div}}_{y^{\prime }}u^{2}+\frac{\partial u_{3}^{1}}{\partial z}=0. \end{array}$$

The boundary conditions are

$$\begin{array}{rl} \epsilon^{0}:u^{0} & =\left\{ \begin{array}{ll} \left(v_{1},v_{2},0\right) & \text{on }\Sigma^{+}\\ 0 & \text{on }\Sigma^{-}\\ \left(g_{1},g_{2},0\right) & \text{on }\Sigma^{w} \end{array}\right.\\ \mathrm{and}\;\;\epsilon^{1}:u^{1} & =\left\{ \begin{array}{ll} \left(0,0,\frac{\partial H}{\partial t}+v\cdot \nabla_{x^{\prime }}H\right) & \text{on }\Sigma^{+}\\ 0 & \text{on }\Sigma^{-}\\ \left(0,0,g_{3}\right) & \text{on }\Sigma^{w} \end{array}\right. \end{array}$$

and with initial conditions

$$\begin{array}{rl} \epsilon^{0}:u^{0} & =(U_{1}^{0},U_{2}^{0},0)\\ \mathrm{and}\;\epsilon^{1}:u^{1} & =(0,0,U_{3}^{0}). \end{array}$$

### (a) Analysis of equations

The main result is as follows.

**Theorem 6.1**

*The leading term u*^0^ *in expansion* (6.1) *for u is given by*

6.9 $$u^{0}=\frac{z(z-H^{*})}{2\nu }\nabla_{x^{\prime }}p^{-2}+\frac{z}{H^{*}}v,$$

*where p*^−2^*, the leading term in expansion* (6.2) *for ϵ*^2^*p, is a solution of the boundary value problem*

6.10 $${\mathrm{div}}_{x^{\prime }}\left(-\frac{{H^{*}}^{3}}{12\nu }\nabla_{x^{\prime }}p^{-2}+\frac{H^{*}}{2}v\right)+\frac{\partial H^{*}}{\partial t}=0\;\text{in }\omega$$

*and*

6.11 $$\left(-\frac{{H^{*}}^{3}}{12\nu }\nabla_{x^{\prime }}p^{-2}+\frac{H^{*}}{2}v-{\bar{g}}^{z*}\right)\cdot \hat{n}=0\;\text{on }\partial \omega .$$

**Proof.**

Multiplying (6.3) with *ϕ*(*y*′)=(*ϕ*_1_,*ϕ*_2_,*ϕ*_3_) such that *ϕ*(*y*′) vanishes near ∂*Y*_*z*_ and integrating over *Y*_*z*_ gives

$$\begin{array}{rl} 0 & =\int_{Y_{z}}-\nu \Delta_{y^{\prime }}u^{0}\cdot \phi +\nabla_{y^{\prime }}p^{-2}\cdot \phi \;dy^{\prime }\\ & =\int_{Y_{z}}\nu \sum_{i=1}^{3}\nabla_{y^{\prime }}u_{i}^{0}\cdot \nabla_{y^{\prime }}\phi_{i}-p^{-2}\;{\mathrm{div}}_{y^{\prime }}\phi \;dy^{\prime }. \end{array}$$

If *z*<H*, then *Y*_*z*_=*Y* and $\partial Y_{z}=\emptyset$ so we can choose *ϕ*=*u*^0^. By (6.6), we obtain

$$0=\int_{Y}\sum_{i=1}^{3}|\nabla_{y^{\prime }}u_{i}^{0}{|}^{2}\;dy^{\prime }⟹\nu \nabla_{y^{\prime }}u^{0}=0\;\text{in }Y;$$

if *z*≥H*, then we can choose $\phi_{i}=u_{i}^{0}-v_{i}.$ Thus,

$$0=\int_{Y_{z}}\sum_{i=1}^{3}|\nabla_{y^{\prime }}u_{i}^{0}{|}^{2}\;dy^{\prime }=0⟹u_{i}^{0}=v_{i}\;\text{in }Y_{z}\;(i=1,2).$$

In both cases, ∇_*y*′_*p*^−2^=0. Summing up, it holds that

6.12a $$\begin{array}{lll} \nabla_{y'}u_{i}^{0} & = & 0\text{ in}\;Y\text{ if}\;z<H*\;(i=1,2,3), \end{array}$$

6.12b $$\begin{array}{rl} u_{i}^{0} & =v_{i}\;\text{in }Y_{z}\;\text{if}z\geq H^{*}\;(i=1,2) \end{array}$$

and

6.12c $$\nabla_{y^{\prime }}p^{-2}=0\;\text{in }B.$$

Integrating (6.7) and using the Gauss–Green theorem gives

$$\begin{array}{rl} 0 & =\int_{B_{z^{\prime }}^{+}}\;{\mathrm{div}}_{y^{\prime }}u^{1}+\frac{\partial u_{3}^{0}}{\partial z}\;dy^{\prime }\;dz\\ & =\int_{\partial B_{z^{\prime }}^{+}}(u_{1}^{1},u_{2}^{1},u_{3}^{0})\cdot \hat{n}\;dS\\ & =\int_{Y_{z^{\prime }}}(u_{1}^{1},u_{2}^{1},u_{3}^{0}){|}_{z=z^{\prime }}\cdot (0,0,-1)\;dy^{\prime }\\ & =-u_{3}^{0}(x^{\prime },z^{\prime },t,\tau )|Y_{z^{\prime }}|. \end{array}$$

Thus, $u_{3}^{0}=0$ for all 0<*z*′<*H*. Thus, (6.4) and 6.7 become

6.13a $$-\nu \Delta_{y^{\prime }}u^{1}+\nabla_{y^{\prime }}p^{-1}+\nabla_{z}p^{-2}=0\;\text{in }B$$

and

6.13b $${\mathrm{div}}_{y^{\prime }}u^{1}=0\;\text{in }B$$

with the boundary conditions

$$u^{1}=\left\{ \begin{array}{ll} \left(0,0,\frac{\partial H}{\partial t}+v\cdot \nabla_{x^{\prime }}H\right) & \text{on }S^{+}\\ 0 & \text{on }S^{-}. \end{array}\right.$$

Multiplying (6.13a) with *ϕ*(*y*′,*z*)=(*ϕ*_1_,*ϕ*_2_,*ϕ*_3_) vanishing on *S*^+^∪*S*^−^ and integrating over B gives

$$\begin{array}{rl} 0 & =\int_{B}\left(-\nu \Delta_{y^{\prime }}u^{1}+\nabla_{y^{\prime }}p^{-1}+\nabla_{z}p^{-2}\right)\cdot \phi \;dy^{\prime }\;dz\\ & =\int_{B}\nu \sum_{i=1}^{3}\nabla_{y^{\prime }}u_{i}^{1}\cdot \nabla_{y^{\prime }}\phi_{i}-p^{-1}\;{\mathrm{div}}_{y^{\prime }}\phi -p^{-2}\frac{\partial \phi_{3}}{\partial z}\;dy^{\prime }\;dz. \end{array}$$

Choosing $\phi =(u_{1}^{1},u_{2}^{1},0)$ gives

$$0=\int_{B}\nu \sum_{i=1}^{2}|\nabla_{y^{\prime }}u_{i}^{1}{|}^{2}\;dy^{\prime }\;dz⟹\nabla_{y^{\prime }}u_{i}^{1}=0\;\text{in }B,\;(i=1,2).$$

Inserting this into (6.13a) yields ∇_*y*′_*p*^−1^=0 in B. The third component of (6.13a) reads

6.14 $$\begin{array}{rl} & -\nu \Delta_{y^{\prime }}u_{3}^{1}+\frac{\partial p^{-2}}{\partial z}=0\;\text{in }B\\ \text{and}\;\; & u_{3}^{1}=\left\{ \begin{array}{ll} \frac{\partial H}{\partial t}+v\cdot \nabla_{x^{\prime }}H & \text{on }\Sigma^{+}\\ 0 & \text{on }\Sigma^{-}. \end{array}\right. \end{array}\}$$

Assume *z*<H*. Integrating (6.14) over *Y* gives

$$0=\int_{Y}-v\Delta_{y'}u_{3}^{1}+\frac{\partial p^{-2}}{\partial z}\text{d}y'=\frac{\partial p^{-2}}{\partial z}|Y|\Rightarrow \frac{\partial p^{-2}}{\partial z}=0\text{ if }0<z<H*$$

Hence,

$$-v\Delta_{y'}u_{3}^{1}=0\text{ in }Y\text{ if }0<z<H*$$

From this we deduce $\nabla_{y^{\prime }}u_{3}^{1}=0.$ Summing up if 0<*z*<H* gives

$$\begin{array}{ll} & \frac{\partial p^{-2}}{\partial z}=0,\text{ if }0<z<H*\\ \text{and} & \nabla_{y'}u_{3}^{1}=0,\text{ if }0<z<H* \end{array}$$

Assume *z*≥H*. As *u*^0^=(*v*_1_,*v*_2_,0) in H*≤*z*<*H*, we deduce from (6.8) that

$${\mathrm{div}}_{y^{\prime }}u^{2}+\frac{\partial u_{3}^{1}}{\partial z}=0\;\text{in }B_{H^{*}}^{+}.$$

So if *z*′>H*, then

$$\begin{array}{rl} 0 & =\int_{B_{H^{*}}^{+}}{\mathrm{div}}_{y^{\prime }}u^{2}+\frac{\partial u_{3}^{1}}{\partial z}\;dy^{\prime }\;dz\\ & =\int_{\partial B_{H^{*}}^{+}}(u_{1}^{2},u_{2}^{2},u_{3}^{1})\cdot \hat{n}\;dS(y^{\prime },z)\\ & =\int_{Y_{z^{\prime }}}(u_{1}^{2},u_{2}^{2},u_{3}^{1})\cdot \left(-\frac{\partial H}{\partial y_{1}},-\frac{\partial H}{\partial y_{2}},1\right)dy^{\prime }\\ & \;+\int_{Y_{z^{\prime }}}(u_{1}^{2},u_{2}^{2},u_{3}^{1}){|}_{z=z^{\prime }}\cdot (0,0,-1)dy^{\prime }\\ & =\int_{Y_{z^{\prime }}}\frac{\partial H}{\partial t}+v\cdot \nabla_{x^{\prime }}H-u_{3}^{1}{|}_{z=z^{\prime }}dy^{\prime }. \end{array}$$

Hence,

6.15 $$\int_{Y_{z}}u_{3}^{1}-\left(\frac{\partial H}{\partial t}+v\cdot \nabla_{x^{\prime }}H\right)dy^{\prime }=0\;\text{for all }z\geq H^{*}.$$

Define $w=u_{3}^{1}-(\partial H/\partial t+v\cdot \nabla_{x^{\prime }}H).$ Then, *w* satisfies

6.16 $$\begin{array}{rl} -\nu \Delta_{y^{\prime }}w+\frac{\partial p^{-2}}{\partial z} & =0\;\text{in }Y_{z}\\ \text{and}\;\;w & =0\;\text{in }\partial Y_{z} \end{array}\}$$

for all *z*≥H*. Multiplying (6.16) with *w* and integrating by parts gives

$$\begin{array}{rl} 0 & =\int_{Y_{z}}\nu |\nabla_{y^{\prime }}w{|}^{2}+\frac{\partial p^{-2}}{\partial z}w\;dy^{\prime }\\ & =\int_{Y_{z}}\nu |\nabla_{y^{\prime }}w{|}^{2}\;dy^{\prime } \end{array}$$

as *p*^−2^ does not depend on *y*′ and

$$\int_{Y_{z}}w\;dy^{\prime }=0$$

by (6.15). Consequently, ∇_*y*′_*w*=0 in $B_{H^{*}}^{+}$, which implies

$$u_{3}^{1}=\frac{\partial H}{\partial t}+v\cdot \nabla_{x^{\prime }}H\;\text{in }B_{H^{*}}^{+}.$$

Assume from now that 0<*z*<H*, equations (6.12a) and (6.12b) imply that *u*^0^ does not depend on *y*′, and (6.5) reduces to

6.17 $$\frac{\partial u^{0}}{\partial \tau }-\nu \left(\frac{\partial^{2}u^{0}}{\partial z^{2}}+\Delta_{y^{\prime }}u^{2}\right)+\nabla_{x^{\prime }}p^{-2}+\nabla_{z}p^{-1}+\nabla_{y^{\prime }}p^{0}=0.$$

Integrating (6.17) over *Y* using also that neither *p*^−2^ nor *p*^−1^ depends on *y*′ gives

$$\frac{\partial u^{0}}{\partial \tau }-v\frac{\partial^{2}u^{0}}{\partial z^{2}}+\nabla_{x'}p^{-2}+\nabla_{z'}p^{-1}=0\text{ if }0<z<H*$$

As $u_{3}^{0}=0,$ this is equivalent to

6.18 $$\frac{\partial u^{0}}{\partial \tau }-v\frac{\partial^{2}u^{0}}{\partial z^{2}}+\nabla_{x'}p^{-2}=0\text{ if }0<z<H*$$

and

6.19 $$\frac{\partial p^{-1}}{\partial z}=0\text{ if }0<z<H*$$

Similarly, integrating (6.8) over *Y* gives

$$0=\int_{Y}{\mathrm{div}}_{x^{\prime }}u^{0}+{\mathrm{div}}_{y^{\prime }}u^{2}+\frac{\partial u_{3}^{1}}{\partial z}\;dy^{\prime }=|Y|\left({\mathrm{div}}_{x^{\prime }}u^{0}+\frac{\partial u_{3}^{1}}{\partial z}\right).$$

Hence,

6.20 $${\mathrm{div}}_{x^{\prime }}u^{0}+\frac{\partial u_{3}^{1}}{\partial z}=0\;\text{in }\Omega^{*}.$$

Consider now (6.18) and (6.20) as a system for $(u^{0},u_{3}^{1},p^{-2}).$ The stationary solution of this system is denoted by $({\hat{u}}^{0},{\hat{u}}_{3}^{1},{\hat{p}}^{-2}),$ which satisfies

$$\begin{array}{rl} -\nu \frac{\partial^{2}{\hat{u}}^{0}}{\partial z^{2}}+\nabla_{x^{\prime }}{\hat{p}}^{-2} & =0\;\text{in }\Omega^{*}\\ \mathrm{and}\;\;{\mathrm{div}}_{x^{\prime }}{\hat{u}}^{0}+\frac{\partial {\hat{u}}_{3}^{1}}{\partial z} & =0\;\text{in }\Omega^{*} \end{array}$$

together with the boundary conditions

$$({\hat{u}}_{1}^{0},{\hat{u}}_{2}^{0},{\hat{u}}_{3}^{1})=\left\{ \begin{array}{ll} \left(v_{1},v_{2},\frac{\partial H^{*}}{\partial t}+v\cdot \nabla_{x^{\prime }}H^{*}\right) & \text{on }{\Sigma^{*}}^{+}\\ 0 & \text{on }{\Sigma^{*}}^{-}\\ \left(g_{1},g_{2},g_{3}\right) & \text{on }{\Sigma^{*}}^{w}. \end{array}\right.$$

We want to show that $(u^{0},u_{3}^{1},p^{-2})=({\hat{u}}^{0},{\hat{u}}_{3}^{1},{\hat{p}}^{-2}).$ To this end, define

$$\begin{array}{rl} {\tilde{u}}^{0} & =u^{0}-{\hat{u}}^{0},\\ {\tilde{u}}_{3}^{1} & =u_{3}^{1}-{\hat{u}}_{3}^{1}\\ \mathrm{and}\;\;{\tilde{p}}^{-2} & =p^{-2}-{\hat{p}}^{-2}, \end{array}$$

which satisfy

6.21 $$\frac{\partial {\tilde{u}}^{0}}{\partial \tau }-\nu \frac{\partial^{2}{\tilde{u}}^{0}}{\partial z^{2}}+\nabla_{x^{\prime }}{\tilde{p}}^{-2}=0\;\text{in }\Omega^{*}$$

and

6.22 $${\mathrm{div}}_{x^{\prime }}{\tilde{u}}^{0}+\frac{\partial {\tilde{u}}_{3}^{1}}{\partial z}=0\;\text{in }\Omega^{*},$$

where ${\tilde{u}}^{0},{\tilde{u}}_{3}^{1}$ and ${\tilde{p}}^{-2}$ are periodic in *τ* and satisfy the boundary conditions

$$({\tilde{u}}_{1}^{0},{\tilde{u}}_{2}^{0},{\tilde{u}}_{3}^{1})=(0,0,0)\;\text{on }{\Sigma^{*}}^{+}\cup {\Sigma^{*}}^{-}\cup {\Sigma^{*}}^{w}.$$

Multiplying (6.21) with *ϕ*=(*ϕ*_1_,*ϕ*_2_,*ϕ*_3_) vanishing on Σ*^+^∪Σ*^−^∪Σ*^w^ and integrating over Ω* gives

$$\begin{array}{rl} 0 & =\int_{\Omega^{*}}\left(\frac{\partial {\tilde{u}}^{0}}{\partial \tau }-\nu \frac{\partial^{2}{\tilde{u}}^{0}}{\partial z^{2}}+\nabla_{x^{\prime }}{\tilde{p}}^{-2}\right)\cdot \phi \;dx^{\prime }\;dz\\ & =\int_{\Omega^{*}}\frac{\partial {\tilde{u}}^{0}}{\partial \tau }\cdot \phi -\nu \frac{\partial {\tilde{u}}^{0}}{\partial z}\cdot \frac{\partial \phi }{\partial z}-{\tilde{p}}^{-2}\;{\mathrm{div}}_{x^{\prime }}\phi \;dx^{\prime }\;dz. \end{array}$$

Choosing $\phi =({\tilde{u}}_{1}^{0},{\tilde{u}}_{2}^{0},{\tilde{u}}_{3}^{1})$ and using (6.22) gives

$$0=\int_{\Omega^{*}}\frac{\partial {\tilde{u}}^{0}}{\partial \tau }\cdot {\tilde{u}}^{0}+\nu {|\frac{\partial {\tilde{u}}^{0}}{\partial z}|}^{2}+{\tilde{p}}^{-2}\frac{\partial {\tilde{u}}_{3}^{1}}{\partial z}\;dx^{\prime }\;dz.$$

As ${\tilde{p}}^{-2}$ does not depend on *z*,

$$\int_{\Omega^{*}}{\tilde{p}}^{-2}\frac{\partial {\tilde{u}}_{3}^{1}}{\partial z}\;dx^{\prime }\;dz=\int_{\omega }{\tilde{p}}^{-2}\int_{0}^{H^{*}}\frac{\partial {\tilde{u}}_{3}^{1}}{\partial z}\;dz\;dx^{\prime }=0.$$

Consequently,

$$\frac{1}{2}\frac{\partial }{\partial \tau }\int_{\Omega^{*}}|{\tilde{u}}^{0}{|}^{2}\;dx^{\prime }\;dz=-\int_{\Omega^{*}}\nu {|\frac{\partial {\tilde{u}}^{0}}{\partial z}|}^{2}\;dx^{\prime }\;dz.$$

Integrating this equality from *τ*=0 to *T* using the periodicity gives

$$0=\int_{0}^{T}\int_{\Omega^{*}}\nu {|\frac{\partial {\tilde{u}}^{0}}{\partial z}|}^{2}\;dx^{\prime }\;dz⟹\frac{\partial {\tilde{u}}^{0}}{\partial z}=0.$$

Hence, ${\tilde{u}}^{0}=0$, which means $u^{0}={\hat{u}}^{0}.$ It follows that $p^{-2}={\hat{p}}^{-2}$ and $u_{3}^{1}={\hat{u}}_{3}^{1},$ so *u*^0^ and *p*^−2^ are independent of *τ*. Hence, equation (6.17) reduces to

6.23 $$-\nu \frac{\partial^{2}u^{0}}{\partial z^{2}}+\nabla_{x^{\prime }}p^{-2}=0\;\text{in }\Omega^{*},$$

with the boundary conditions

$$u^{0}=\left\{ \begin{array}{ll} \left(v_{1},v_{2},0\right) & \text{on }{\Sigma^{*}}^{+}\\ 0 & \text{on }{\Sigma^{*}}^{-}\\ \left(g_{1},g_{2},g_{3}\right) & \text{on }{\Sigma^{*}}^{w}. \end{array}\right.$$

Integrating with respect to *z* gives (6.9). Integrating again (6.9) with respect to *z* gives

6.24 $${\bar{u^{0}}}^{z*}=-\frac{{H^{*}}^{3}}{12\nu }\nabla_{x^{\prime }}p^{-2}+\frac{H^{*}}{2}v.$$

In similar fashion as above (6.20) gives

6.25 $${\mathrm{div}}_{x^{\prime }}{\bar{u^{0}}}^{z*}+\frac{\partial H^{*}}{\partial t}=0\;\text{in }\omega$$

and

6.26 $$\left({\bar{u^{0}}}^{z*}-{\bar{g}}^{z*}\right)\cdot \hat{n}=0\;\text{on }\partial \omega .$$

Inserting (6.24) into (6.25) and (6.26), we obtain the classical Reynolds equation (6.10), in ‘minimum’ film thickness H*, with the boundary condition (6.11). □

**Remark 6.2**

The equations of theorem 6.1 can also be obtained by letting λ→∞ in the equations of theorem 4.1.

## 7. Summary and conclusion

As mentioned in the Introduction, three flow regimes have been identified,

(1) Stokes roughness, 0<λ<∞ (see theorem 4.1),
(2) Reynolds roughness, λ=0 (see theorem 5.1), and
(3) high-frequency roughness regime, λ=∞ (see theorem 6.1).

In all three cases, we end up with a two-dimensional equation of the form

$$\begin{array}{rl} \mathrm{div}(A^{\lambda }\nabla p+b^{\lambda })+\frac{\partial h_{0}}{\partial t} & =0\;\text{in }\omega \times (0,T]\\ \mathrm{and}\;\;(A^{\lambda }\nabla p+b^{\lambda }-{\bar{g}}^{z})\cdot \hat{n} & =0\;\text{on }\partial \omega \times (0,T], \end{array}$$

where *p* is the unknown pressure, ∇=(∂/∂*x*_1_,∂/∂*x*_2_) and div=∇⋅,

$$A^{\lambda }=\left(\begin{array}{cc} a_{11} & a_{12}\\ a_{21} & a_{22} \end{array}\right)\;\text{and}\;b^{\lambda }=\left(\begin{array}{c} b_{1}\\ b_{2} \end{array}\right).$$

The matrix *A*^λ^ and vector *b*^λ^ are macroscopic quantities known as ‘flow factors’. They are calculated by solving local problems on a periodic cell, thus taking into account the local geometry, i.e. the roughness, of the problem. The expression *A*^λ^∇*p*+*b*^λ^ comes from averaging the first two components of the velocity field ${\bar{{\bar{u}}^{z}}}^{y^{\prime }}.$ As the flow is governed by an equation which is a generalized form of the Reynolds equation, one can say that the thin film approximation is valid on the macroscopic scale in all three cases. In the Stokes roughness regime, the thin film approximation is not valid on the microscopic scale—the local problems are periodic analogues of the Stokes equation and three dimensional. Consequently, the calculation of the flow factors comes at a high cost. However, as λ tends to zero, the solution of the local problems asymptotically satisfies problems that are local variants of the classical Reynolds equation. Thus, the thin film approximation is valid also on the microscopic level in the Reynolds roughness regime. In conclusion, one can say that some information about the flow on the microscopic level is lost at the extreme cases λ=0 and λ=∞. In fact, $A^{\infty }$ and $b^{\infty }$ retain no information about the roughness (except the minimum height) and the cell problems have trivial solutions. The limiting equation in the high-frequency regime is exactly the classical Reynolds equation, which has been well studied. It can be interpreted as though the flow is prevented from entering the thin valleys of the rough surfaces. The information loss in the case λ=0 is due to the thin film approximation on the microscopic level. It would be interesting to compare *A*^λ^, *b*^λ^ to *A*^0^,*b*^0^ for small values of λ as well as the corresponding flow patterns. We hope to accomplish such a study in the future including numerical simulations.

As to previous studies, the present result reduces to the stationary case when ∂*h*_0_/∂*t*=0 and ∂*h*_per_/∂*τ*=0. Compare with eqn (17) in [4] and theorem 3.1 in [11] for the Stokes roughness; eqn (25) in [4] and theorem 3.2 in [11] for the Reynolds roughness; and eqn (20) in [4] for the high-frequency roughness. Note that, in the unstationary case, time plays only the role of a parameter in all three limiting equations. Although the original equation (2.1) contains the term ∂*u*/∂*t*, the time derivative of the unknown solution does not appear in the limiting equation nor in the local problems.
